# Delineation of prostatic calcification using quantitative susceptibility mapping: Spatial accuracy for magnetic resonance‐only radiotherapy planning

**DOI:** 10.1002/acm2.13469

**Published:** 2021-11-02

**Authors:** Hirohito Kan, Takahiro Tsuchiya, Masato Yamada, Hiroshi Kunitomo, Harumasa Kasai, Yuta Shibamoto

**Affiliations:** ^1^ Department of Integrated Health Sciences Graduate School of Medicine Nagoya University Nagoya Japan; ^2^ Department of Radiology Graduate School of Medical Sciences Nagoya City University Nagoya Japan; ^3^ Department of Radiology Nagoya City University Hospital Nagoya City University Nagoya Japan

**Keywords:** image‐guided radiotherapy, MR‐only simulation, quantitative susceptibility mapping

## Abstract

To investigate the spatial accuracy of delineating prostatic calcifications by quantitative susceptibility mapping (QSM) in comparison with computed tomography (CT), we conducted phantom and human studies. Five differently‐sized spherical hydroxyapatites mimicking prostatic calcification (pseudo‐calcification) were arranged in the order of their sizes at the center of a plastic container filled with gelatin. This calcification phantom underwent magnetic resonance (MR) imaging, including the multiple spoiled gradient‐echo sequences (SPGR) for the QSM and CT as a reference. The volume of each pseudo‐calcification and center‐to‐center distance between the pseudo‐calcifications delineated by QSM and CT were measured. In the human study, eight patients with prostate cancer who underwent radiation therapy and had some prostatic calcifications were included. The patients underwent CT and SPGR and modified DIXON sequence for MR‐only simulation. The hybrid QSM processing combined with the complex signals in the SPGR and water and fat fraction maps estimated from the modified DIXON sequence were used to reconstruct the pelvic susceptibility map in humans. The threshold of CT numbers was set at 130 HU, while the QSM images were manually segmented in the calcification phantom and human studies. In the phantom study, there was an excellent agreement in the pseudo‐calcification volumes between QSM and CT (*y* = 1.02*x* – 7.38, *R* ^2^ = 0.99). The signal profiles had similar trends in CT and QSM. The center‐to‐center distances between the pseudo‐calcifications in the phantom were also identical in QSM and CT. The calcification volumes were almost identical between the QSM and CT in the human study (*y* = 0.95*x* – 9.32, *R* ^2^ = 1.00). QSM can offer geometric and volumetric accuracies to delineate prostatic calcifications, similar to CT. The prostatic calcification delineated by QSM may facilitate image‐guided radiotherapy in the MR‐only simulation workflow.

## 1 INTRODUCTION

Intensity‐modulated radiotherapy that can deliver high doses to the target while reducing the doses to the surrounding risk organs is an established treatment modality for prostate cancer. Image‐guided radiotherapy (IGRT) is essential to accurately deliver radiation doses to prostate cancer and reduce the risk of complications in the rectum and bladder. Computed tomography (CT) plays a key role in calculating the delivery dose to the prostate and performing IGRT. As CT allows good delineation of calcification, the delineated prostatic calcification is often utilized as a natural fiducial marker for IGRT in patients without implanted fiducial markers. Hanna et al.^1^ reported that IGRT using prostatic calcification as a marker had adequate precision.

Recently, a commercial software has been released for magnetic resonance (MR)‐only treatment planning. The MR‐only simulation produces pseudo‐CT images using gradient‐echo images for the modified DIXON (mDIXON) method.^2^, ^3^ The pseudo‐CT is generated based on the segmentation results of the mDIXON images. Therefore, the image contrast in pseudo‐CT is limited to only five classes: air, soft tissue, fat, spongy bone, and compact bone. It implies that the image contrast between the prostate and risk organs classified as soft tissue disappears. Kan et al.^4^ showed that the accuracy of IGRT using the pseudo‐CT alone was insufficient in patients without implanted fiducial markers. Therefore, techniques to accurately delineate prostatic calcification are desired in MR‐only simulation for performing IGRT, because calcifications have no MR signals on a conventional pulse sequence.

Quantitative susceptibility mapping (QSM) is an advanced MR imaging (MRI) technique that enables direct estimation of magnetic susceptibility from phase images of the gradient‐echo sequence.^5^ As the diamagnetic calcification represents negative susceptibility, the QSM may be a promising novel technique for the depiction of calcification in MRI. In this study, we compared the spatial accuracy of the delineated prostatic calcification between CT and QSM using a phantom and actual patients, and investigated whether the prostatic calcification delineated by QSM can be utilized as a fiducial marker in IGRT.

## 2 METHODS

### 2.1 Phantom preparation

**FIGURE 1:**
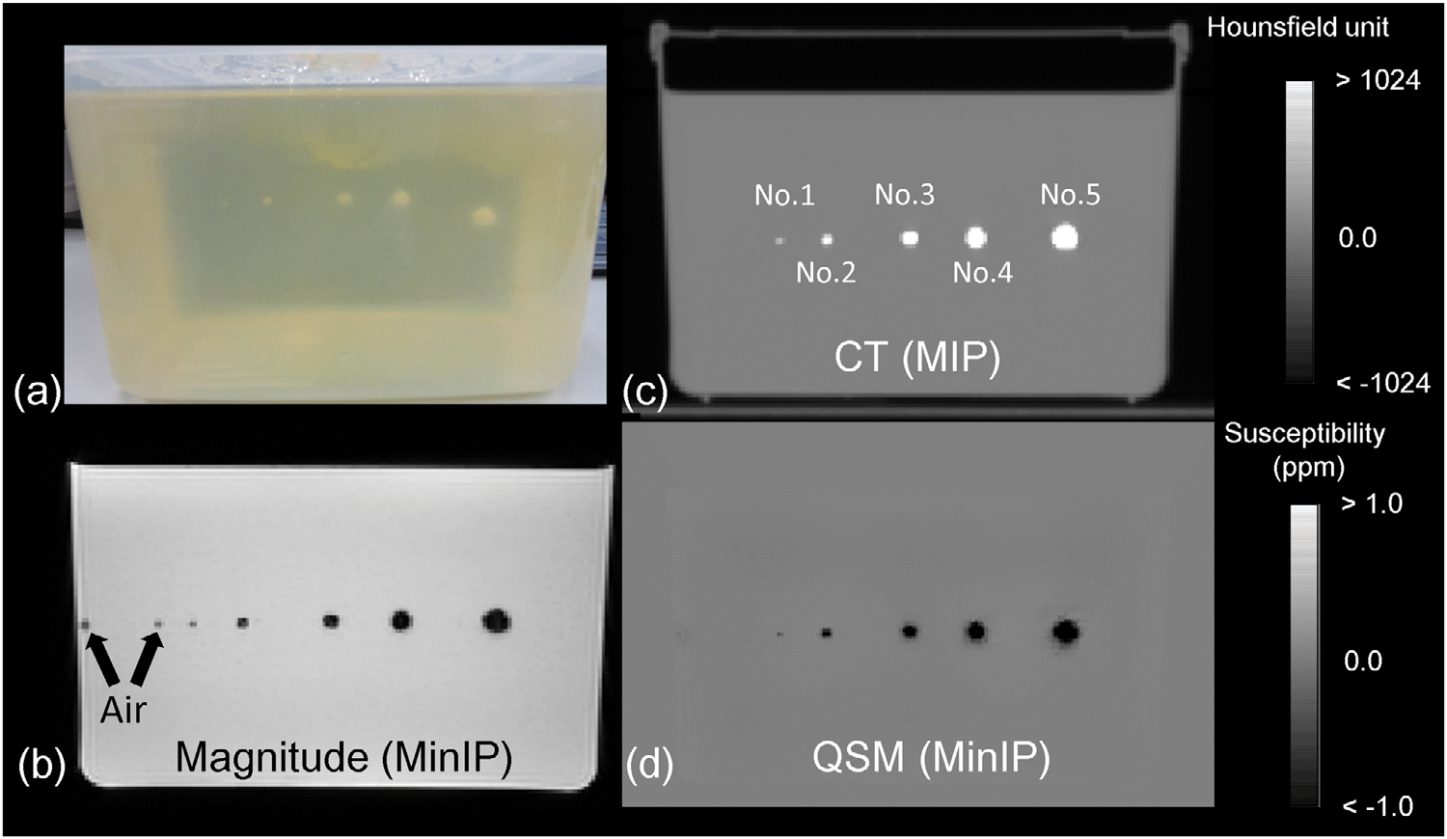
(a) Lateral view of a calcification phantom. Pseudo‐calcifications No. 1–5 were arranged from the left in ascending order. (b) Magnitude image of the first echo in multiple spoiled gradient‐echo sequences that underwent minimum intensity projection (MinIP). The air was visualized as small dots. (c) Computed tomography images that underwent maximum intensity projection. (d) Susceptibility map estimated by quantitative susceptibility mapping reconstruction that underwent MinIP

The phantom study aimed to compare the geometric and volumetric accuracies of delineating calcification‐like materials by QSM and CT. Five differently‐sized spherical hydroxyapatites mimicking prostatic calcification (pseudo‐calcification) were arranged in the order of their sizes at the center of a plastic container (120 × 160 × 110 mm) filled with gelatin. The radii of hydroxyapatites were changed by approximately 1–5 mm (Figure 1a). The low signal areas around the hydroxyapatite were air cavities in the magnitude image of multiple spoiled gradient‐echo sequences (SPGR) (Figure 1b). The calcification phantom was placed at the center of a gantry in MRI and its images were obtained. CT images were also acquired as a reference (Figure 1c). The SPGR was acquired to reconstruct the susceptibility map (Figure 1d).

### 2.2 Human study

This retrospective study was approved by the institutional review board. We included eight patients with prostate cancer who underwent intensity‐modulated radiotherapy using MR‐only simulation between February and October 2019. The inclusion criteria were the presence of prostatic calcifications and no history of surgery. Before image acquisitions in each patient, the vacuum cushion for the whole pelvis was made to improve reproducibility of the treatment position. Sufficient urine collection and lack of excessive feces and gas in the rectum were confirmed. The planning CT, SPGR, and pseudo‐CT for MR‐only simulation were routinely performed on the same day. Whole pelvis images were acquired by CT, covering the full body contour in the axial plane. Next, the SPGR and mDIXON for pseudo‐CT which are commercially available were acquired. To reproduce the patient's position between the planning CT and pseudo‐CT, the same vacuum cushion and anterior coil support were used in MRI acquisition. The time gap between CT and MRI acquisitions was 1 h because the patients were requested to urinate to maintain the same conditions of urine collection between the scans.

### 2.3 Image acquisition

Data acquisitions were conducted on Optima 580 (Milwaukee, WI, USA) and Ingenia 1.5T RTsim (Philips, Best, the Netherlands). The CT and SPGR images were acquired in the phantom and human studies. The mDIXON images for pseudo‐CT were additionally acquired in the human study.

The scan parameters for CT were as follows: field of view, 500 × 500 mm; matrix size, 512 × 512; slice thickness, 2.5 mm; and tube voltage, 120 kV. The detailed parameters of SPGR for QSM were as follows: field of view, 384 × 384 × 125 mm; matrix size, 384 × 384 × 50; slice thickness, 2.5 mm; echo time (TE), 5.7–40.7 ms at 7.0 ms interval; number of echoes, 6; repetition time (TR), 45.0 ms; flip angle, 20°; and parallel imaging factor, 2.5. The total scan time was 349 s. Scan parameters of mDIXON images were: field of view, 546 × 546 × 120 mm; matrix size, 320 × 321 × 120; slice thickness, 2.5 mm; TE, 1.78 and 4.0 ms; and TR, 6.0 ms. The pseudo‐CT image was automatically reconstructed on an imaging console from mDIXON images using in‐phase images and water and fat fraction maps.

### 2.4 Quantitative susceptibility mapping

QSM reconstruction requires three‐step processing: phase unwrapping, background field removal, and dipole inversion. In the phantom study, the multiple‐phase images were unwrapped by 3D Laplacian‐based phase unwrapping.^6^ The Laplacian boundary problem method^7^ was applied in the unwrapped phase images, followed by variable kernels sophisticated harmonic artifact reduction for phase image with kernel sizes^8^ from 1 to 25 mm at 2‐mm intervals to eliminate the background field. The fourth‐order polynomial fitting was performed in multiple echo images.^9^ To compute the local field while being effectively combined with the image of each echo, the T_2_* map‐based weighted average was performed.^10^ The susceptibility map was reconstructed from the local field map by the improved sparse linear equations and least squares algorithm.^11^

In the human study, the magnitude and phase images in fat regions were modulated by a chemical shift of fat, that is, 3.5 ppm,^12^ expressed by Equation (1):

(1) $$S_{i}=M_{0}\left(W+Fe^{i2\pi f_{f}TE_{i}}\right)e^{-R_{2}^{*}TE_{i}}e^{i2\pi \varphi TE_{i}}$$

where *S_i_* is the complex signal in *i*th *TE*; *M*_0_, *W*, and *F* are net magnetization and fractions of water and fat, respectively; *f_f_* is phase‐frequency induced by the chemical shift of fat; R_2_* is reciprocal of T_2_* value; and *φ* is total field map summed tissue from the local and background fields. It is difficult to estimate water‐fat separation from the original complex signals in the SPGR in this study because of the relatively long echo spacing. The water and fat fractions estimated by mDIXON images for pseudo‐CT were utilized to correct the effect of the fat chemical shift from the complex signals. The water and fat fraction maps in the mDIXON images were co‐registered to the magnitude image of the first echo in SPGR and resliced to its magnitude image using Statistical Parametric Mapping 12 (https://www.fil.ion.ucl.ac.uk/spm/). These co‐registered and resliced water and fat fraction images were substituted to the *W* and *F* in Equation (1). Then, Equation (1) was converted into Equation (2).

(2) $$\begin{array}{c} \ln S_{i}=A+BTE_{i}\\ A=lnM_{0}\left(W+Fe^{i2\pi f_{f}TE_{i}}\right),\;B=-R_{2}^{*}+i2\pi \varphi \end{array}$$

This linear equation with complex numbers was solved. *M*_0_ and *R*2* were estimated using A and B, expressed to Equations (3) and (4):

(3) $$M_{0}=\frac{\exp\left(A\right)}{\left(W+Fe^{i2\pi f_{f}TE_{i}}\right)}$$

(4) $$R_{2}^{*}=-real\left(B\right)$$

Using the estimated *M_0_*, *W*, *F*, and *R*_2_ * were substituted in Equation (1) to calculate the phase values at each TE correcting the fat chemical shift. The calculated phase images of the first three echoes were processed by the QSM processing aforementioned in the phantom study. The QSM reconstruction algorithm used in this study was written by MATLAB R2020b (MathWorks Inc., Natick, MA, USA). It took about 30 min for susceptibility map calculation in this study.

### 2.5 Spatial accuracy of calcification

In the phantom study, to validate the spatial accuracy of pseudo‐calcifications estimated by QSM, we measured the volumes of the pseudo‐calcifications, the center‐to‐center distance between the pseudo‐calcifications, and full width at half maximum (FWHM) at a maximal diameter of the delineated pseudo‐calcifications in QSM and CT. First, spatial registration between CT and susceptibility map was performed based on the MR‐visible markers on the outer wall of a plastic container using the registration function in ITK‐SNAP.^13^ The threshold of CT numbers was set at 130 HU based on the previous study,^14^ while the QSM images were manually segmented in the calcification phantom to determine the 3D volume of interests using ITK‐SNAP. The signal profiles at each pseudo‐calcification and FWHM in their profiles were determined. Moreover, the center‐to‐center distance between the pseudo‐calcifications was also determined manually.

In the human study, the threshold of CT numbers was also set at 130 HU to measure the delineated total volume of prostatic calcifications in each patient.^14^ The total prostatic volume delineated by QSM was manually determined using the ITK‐SNAP.

To assess the interobserver agreement, manual segmentations of prostatic calcification in the QSM were performed by two expert radiographers (8 and 10 years of experience). Wilcoxon signed‐rank test was used to assess volumes, FWHM, and center‐to‐center distance in the phantom study. Spearman's rank correlation analysis was used to evaluate the volumes between CT and QSM and interobserver agreement for the volumes in QSM.

## 3 RESULTS

### 3.1 Phantom study

**FIGURE 2:**
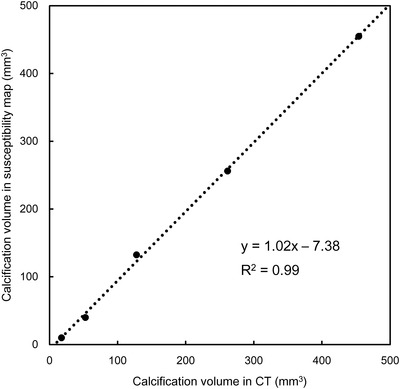
Relationship between the delineated pseudo‐calcification volumes in computed tomography (CT) and quantitative susceptibility mapping (QSM) analysis in the calcification phantom. There was a strong positive correlation, and the measured volumes in both coincided well

**FIGURE 3:**
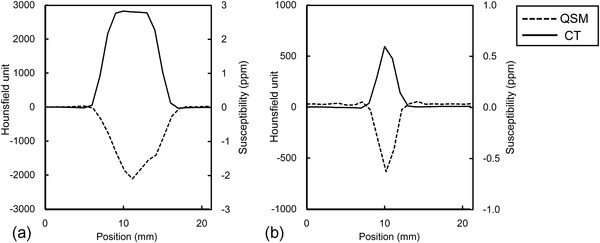
Signal profiles of pseudo‐calcification (a) No. 5 and (b) No. 2 delineated by computed tomography (CT) and quantitative susceptibility mapping (QSM) analysis

There was an excellent agreement in the pseudo‐calcification volumes between QSM and CT (*y* = 1.02*x* – 7.38, *R* ^2^ = 0.99) (Figure 2). Figure 3 shows the representative signal profiles in the second and fifth pseudo‐calcifications. They had similar trends in CT and QSM, although those FWHMs in QSM were significantly smaller than those in CT (*p* < 0.01) (Table 1). Conversely, the center‐to‐center distances between the pseudo‐calcifications were almost identical in QSM and CT (Table 2).

**TABLE 1:** Full width at half maximum in each delineated pseudo‐calcification

	**No. 1**	**No. 2**	**No. 3**	**No. 4**	**No. 5**
QSM	1.59	2.17	3.64	4.41	6.13
CT	2.30	2.51	3.96	4.89	7.28

Abbreviations: CT, computed tomography; QSM, quantitative susceptibility mapping.

**TABLE 2:** Center‐to‐center distance between neighboring pseudo‐calcifications

	**Distance from No. 5 to No. 4(mm)**	**Distance from No. 4 to No. 3(mm)**	**Distance from No. 3 to No. 2 (mm)**	**Distance from No. 2 to No. 1(mm)**
QSM	27.6	20.4	25.4	15.1
CT	27.5	20.4	25.4	15.3

Abbreviations: CT, computed tomography; QSM, quantitative susceptibility mapping.

### 3.2 Human study

**FIGURE 4:**
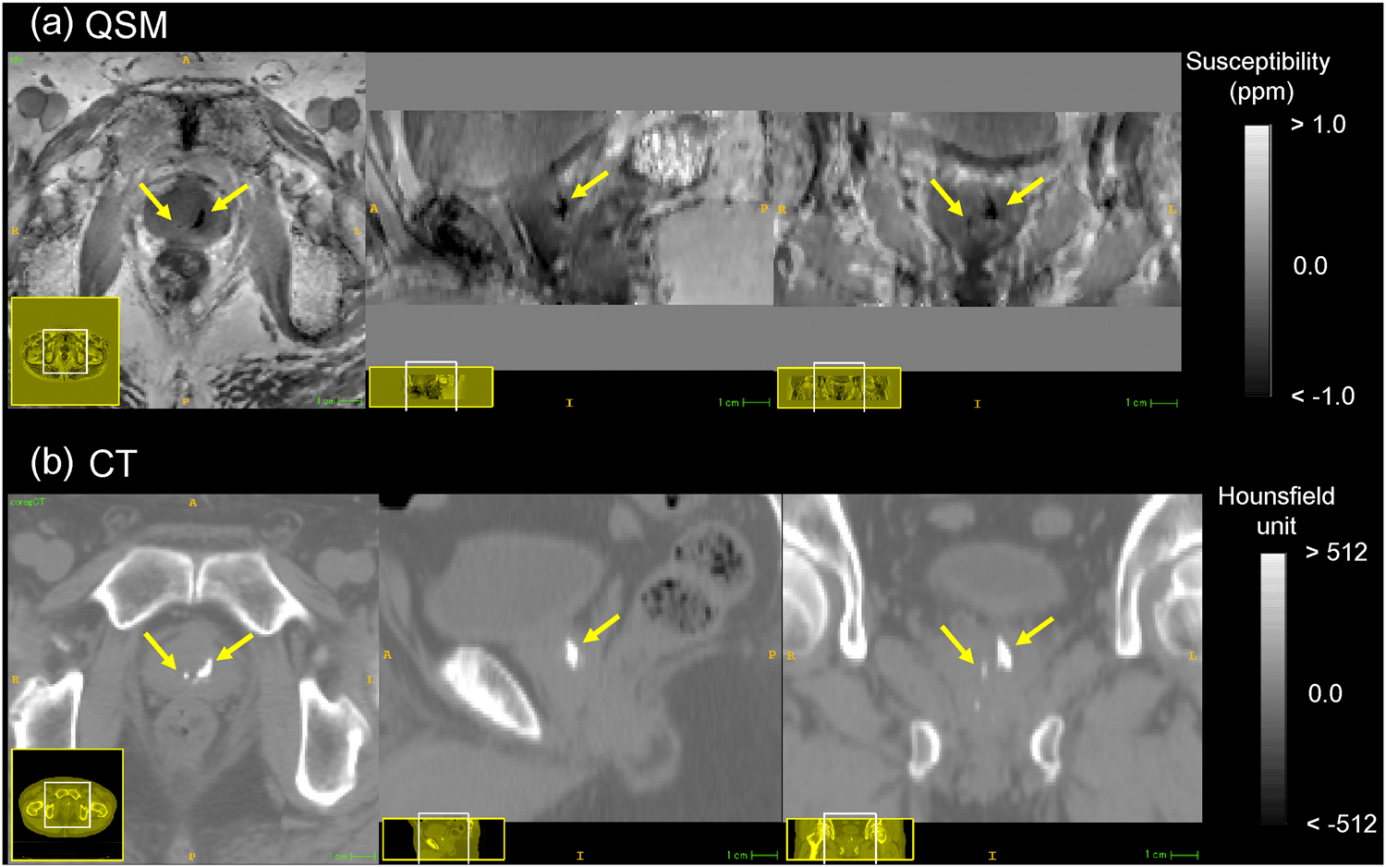
Representative prostatic calcifications (yellow arrow) in (a) quantitative susceptibility mapping and (b) computed tomography

**FIGURE 5:**
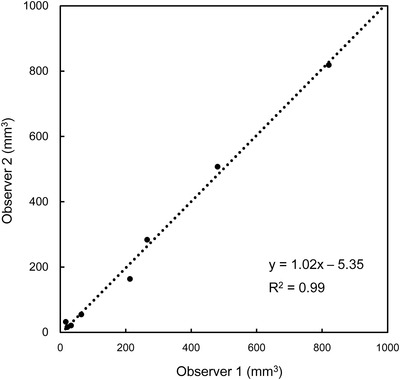
Regression analysis in the segmented calcification volume between the two observers in patients with prostate cancer. The volumes in two observers coincided well

**FIGURE 6:**
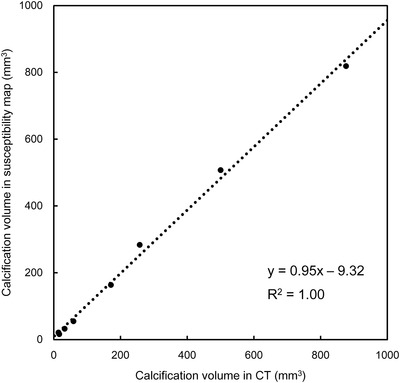
Relationships in the segmented volumes between the quantitative susceptibility mapping and computed tomography in patients with prostate cancer

The susceptibility map successfully delineated the calcification in the prostate using our QSM reconstruction algorithm (Figure 4). The calcification volumes segmented by two observers had a good agreement (*y* = 1.02*x* – 5.35, *R* ^2^ = 0.99) (Figure 5). The calcification volumes had excellent agreements between the QSM and CT (*y* = 0.95*x* – 9.32, *R* ^2^ = 1.00) (Figure 6).

## 4 DISCUSSION

In the present study, we performed phantom and human studies to investigate the geometric and volumetric accuracies in the calcification delineation by using QSM analysis in comparison with those obtained by CT as a reference. The important finding was that the segmented volumes of pseudo‐calcification in the QSM excellently corresponded to the pseudo‐calcification volumes in the CT in the phantom study. This result was clearly explained by signal profiles in the QSM demonstrating a similar fashion to that in CT (Figure 2). The origin of the sidelobe in the signal profiles was close to equivalent, indicating the CT and QSM were not differentiated in the calcification delineation. Moreover, the result of the center‐to‐center distance between the pseudo‐calcifications showed that the positions in the calcification phantom were accurately depicted in QSM and CT. This geometric correspondence was already reported by Nosrati et al.,^15^ who revealed that the QSM allows visualization of the calcification and may have the potential to replace CT. However, the FWHM results in QSM were slightly but significantly smaller than those in CT. This is because there was a difference in the profile shape between CT and QSM. The signal profile in CT represented the plateau around the center of calcification, while the signal profile in QSM showed a sharp shape. Calcifications are small deposits of calcium. The calcium readily absorbs X‐rays from CT. However, the calcifications cannot typically be delineated by conventional MRI. Bai et al.^16^ reported that the susceptibility‐weighted image (SWI) could detect prostatic calcification. SWI allows visualization of the calcification because SWI utilizes the high‐pass‐filtered phase, which reflects the magnetic property well. However, the calcification diameter and volume may be overestimated due to the blooming artifact in the SWI. Additionally, SWI contrast is affected by the TE setting, main field strength, and high‐pass filter size. Consequently, the visualization of calcification is changed. Conversely, the QSM can offer a spatial accuracy of the visualized calcification in MRI, as shown in our result, and might be a more appropriate way to depict calcification than SWI and other MR sequences.

In the human studies, our hybrid reconstruction algorithm combined with pre‐prepared water and fat fraction maps and complex image signals in SPGR could delineate the prostatic calcification well and generally reconstruct the good pelvic susceptibility map. The manual segmentation volumes of calcification between two experts coincided well. The reason for the use of manual segmentation was that the susceptibility map had a slight shading artifact due to the larger field inhomogeneity induced by the rectum and no reference regions of susceptibility value were set in this study. For these reasons, the threshold of susceptibility value could not be uniquely set. The volume measurement of the prostatic calcification in the QSM corresponded to that in CT. The QSM provided a geometrically accurate estimation from phase evolution around it through field‐source inversion.^17^ This is because the effects of phase modulation due to the susceptibility of source (i.e., calcification) and dipole interaction between it and surrounding tissues are minimized by the dipole inversion process in QSM analysis compared with other MR sequences. Therefore, the QSM may have an identical ability to delineate the prostatic calcifications to CT besides offering the quantitative value.^18^ Their geometric and volumetric accuracies in the QSM might be available in IGRT based on the prostatic calcification delineated by the QSM as a natural fiducial marker in patients without a fiducial marker. To the best of our knowledge, this is the first study that deals with the segmented calcification volumes between the QSM and CT in vivo. Moreover, because the QSM analysis serves the contrast depending on the magnetic property, the implanted fiducial markers can be detected besides a natural marker such as calcification. Therefore, the QSM analysis can be applied for even the other organs performed IGRT. For the CT‐MR registration even in the online MR‐linac system, the delineation of prostatic calcification may allow improving IGRT accuracy.

There were some limitations to this study. Although the QSM reconstruction algorithm we used in the human study was combined with SPGR and water and fat fraction maps estimated from the mDIXON sequence, the simultaneous water‐fat separation, and QSM estimation is more appropriate using a single SPGR acquisition according to the previous study.^19^ However, simultaneous estimation could not be performed because of improper echo spacing in the SPGR. Moreover, the shading artifact occurred in the reconstructed susceptibility map. Further studies are needed to perform simultaneous estimations of water‐fat separation and QSM, minimization of shading artifact, and parameter optimization of SPGR. The reason for the use of manual segmentation of calcification in the QSM was the shading artifact described above. Ideally, it is desirable that the specific threshold of susceptibility is set to segment the prostatic calcification.

## 5 CONCLUSION

QSM and CT can offer geometric and volumetric accuracies to delineate prostatic calcifications. The prostatic calcification delineated by QSM may be utilized for IGRT in the MR‐only workflow.

## CONFLICT OF INTEREST

The authors declare that they have no conflict of interest.

## AUTHOR CONTRIBUTIONS

Hirohito Kan: Conceptualization, methodology, software, formal analysis, investigation, data curation, writing‐ original draft, and funding acquisition. Takahiro Tsuchiya: Formal analysis, investigation, data curation, supervision, resources, writing, review, and editing. Masato Yamada: Data curation and investigation. Hiroshi Kunitomo: Data curation and investigation. Harumasa Kasai: Supervision, writing, review, and editing. Yuta Shibamoto: Supervision, writing, review, and editing.
